# The best choices: the diversity and functions of the plants in the home gardens of the Tsang-la (Motuo Menba) communities in Yarlung Tsangpo Grand Canyon, Southwest China

**DOI:** 10.1186/s13002-020-00395-z

**Published:** 2020-08-31

**Authors:** Yu Zhang, Li-Xin Yang, Ming-Xiang Li, Yong-Jie Guo, Shan Li, Yu-Hua Wang

**Affiliations:** 1 Yunnan Key Laboratory for Wild Plant Resources, Kunming Institute of Botany, Chinese Academy of Sciences, Kunming, China; 2 Key Laboratory of Economic Plants and Biotechnology, Kunming Institute of Botany, Chinese Academy of Sciences, Kunming, China; 3 University of Chinese Academy of Sciences, Beijing, China; 4 Germplasm Bank of Wild Species of China, Kunming Institute of Botany, Chinese Academy of Sciences, Kunming, 650201 Yunnan China; 5 Key Laboratory for Microbial Resources of the Ministry of Education, Yunnan Institute of Microbiology, School of Life Sciences, Yunnan University, Kunming, 650091 China; 6 State Key Laboratory of Photochemistry and Plant Resources in West China, Kunming Institute of Botany, Chinese Academy of Sciences, Kunming, China

**Keywords:** Home garden, Local knowledge, Yarlung Tsangpo Grand Canyon, Tibetan, Southwest China

## Abstract

**Background:**

Home garden is identified as a kind of small-scale land-use system which is used to manage and cultivate useful plants by local people, and home gardens can provide various plant products and services. Investigating home gardens was regarded as an effective way to understand the biodiversity-related local knowledge and culture of native people in Ethnobiology and Ethnoecology. Home garden is important in less developed and remote areas. The grand canyon of Yarlung Tsangpo is designed as one of the biodiversity hotspots of China, and it is one of the most remote regions of China, because of the rough traffic conditions. The aim of the present study is to collect, record, and document the plants and their local knowledge and functions in the local home gardens, then attempt to answer the question: “why local people selected these plants?”

**Material and methods:**

The study area was in Beibeng Township of Motuo County in the grand canyon of Yarlung Tsangpo. Observation and semi-structure interviews with informed consent were used to collect data in field study. All information was collected and organized, then documented based on “ethno-species” as a fundamental unit. All of the information of local use and knowledge were organized as the list of “use-report” for quantitative analysis, and the local uses of plants were merged into 14 use categories. Frequency of citation (FC), relative frequency of citation (RFC), cultural importance index (CI), and cultural value index (CV) were used in quantitative analysis. Besides, the Jaccard Index was used to compare the similarity in plant species selection among different communities.

**Results:**

A total of 78 home gardens in the 9 communities of Beibeng Township were visited, and 196 ethno-species were collected. These ethno-species were identified into 188 Botanical taxa. A total of 87 home garden owners as informants were interviewed in the present study, and they provided 625 use-reports to us. The top 5 important plants were Su-lan-tsao (*Dendrobium nobile*), Sa-ga (*Zingiber officinale*), Soe-lu (*Capsicum annuum*), Snying-pa (*Citrus medica*), and Kham-pu (*Prunus persica*), according to the quantitative analysis. The most citied use-category was “vegetable,” followed by “ornamental plant,” “medicine,” and “fruit.” The altitude might be the most important impact factor of the plant diversity and composition of home gardens, and the traffic conditions, local terrain, also impact the plant diversity and composition of home gardens.

**Conclusion:**

In remote areas such as the Yarlung Tsangpo Grand Canyon, the plants in home gardens are important sources of plant products such as foods, herbal medicines, and fibers to support daily lives. The local home gardens in Tsang-la communities had high diversity of plants, and these plants provided many functions and services to support daily lives of local people. Local plant knowledge, including the features, life forms, habits, habitats, and use values of plants, were the summary of the understanding of local people to their surrounding plant worlds. Local people selected appropriate plants to cultivate and manage in their home gardens under the guidance of the local plant knowledge. That is the answer to the question “why local people selected these plants?”

## Background

Home garden is identified as a kind of small-scale land-use system which is used to manage and cultivate useful plants by local people, and home gardens can provide various plant products and services [1–7]. Besides, the economic plants cultivated in home gardens can bring extra income to local people [3]. In some parts of the world, home gardens also have important social and cultural significances [4]. Thus, investigating home gardens was regarded as an effective way to understand the biodiversity-related local knowledge and culture of native people in Ethnobiology and Ethnoecology. In remote areas, the plants in home gardens are important sources of plant products such as foods, herbal medicines, and fibers to support daily lives [1–12]. Some previous studies in less developed areas indicated that most of the nutrients of local people, such as vitamins and minerals, were from the vegetables and fruits in home gardens [3, 9]. In home gardens, the plant products which were rich in starch can help local people to fight against famine in remote areas [9–13]. Therefore, home garden is important in less developed and remote areas.

Locate in the core region of the Himalaya biodiversity hotspot, the grand canyon of Yarlung Tsangpo is designed as one of the national natural reserves of China [14, 15]. As one of the deepest and longest canyons in the world, Yarlung Tsangpo have rich diversity of habitats for its huge elevation drop. Thousands of plants and animals grow here. Thus, the grand canyon of Yarlung Tsangpo was known as “the museum of vegetation” and “the paradise of lives” [16, 17]. Two counties including Milin (Smin-gling Rdzong, 米林县) and Motuo (Me-dog Rdzong, 墨脱县) belong to Linzhi Prefecture (Nying-khri Krong, 林芝市) of Xizang Autonomous Region locate in the grand canyon, and several Chinese ethnic groups settle in the region. Gongbo Tibetan, Tsang-la, and Lho-pa people is the main population of the region. They have accumulated rich local knowledge of their surrounding natural world [18]. However, people who live in the region have to face the difficulties of transport caused by the rough terrain, which limits the exchanges of products and knowledge between local communities and outside. For example, Motuo County which were deep in the grand canyon had no complete motor way to outside until 2013, and some townships of the county even had no motor way until early 2018 [17]. Motuo County had been one of the most backward regions of China, because of the terrible transport condition. Therefore, the region became one of the key regions of the poverty alleviation program of China [18].

Most of previous studies in the region were about biodiversity, and just few studies involved the relationship between local people and biodiversity such as the local knowledge of plants [16]. In our preliminary Ethnobotanical studies of local people in the region, we found rich local knowledge of plants [19–21]. The functions of the plants in home gardens played important roles in the daily lives of local people. And some previous studies in the of Himalaya area outside China showed rich diversity and local knowledge of plants in traditional home gardens [22–24]. Thus, it is necessary to investigate the home gardens of local communities in the grand canyon of Yarlung Tsangpo.

Tsang-la people settle in the Yarlung Tsangpo basin of Xizang Autonomous Prefecture of China [25]. The Tsang-la language belongs to the Tibetan Language Branch of Chinese-Tibetan Language Family. Because the ancestors of Tsang-la people originated from the “Menyu (门隅)” District of Tibetan Plateau, they were also identified as “Motuo Menba (墨脱门巴),” a branch of the Chinese Menba nationality (门巴族). “Motuo Menba” means “Menba people who live in Motuo County.” The history of Tsang-la people settling in Motuo County could be traced to over 300 years ago (early Qing Dynasty of China). Therefore, Tsang-la people were also called as “Pad-ma-rkos-pa” in Tibetan language, which meant “people of Pad-ma-rsko.” “Pad-ma-rkos (白玛岗)” was the ancient name of Motuo County in Qing Dynasty of China [25–28].

As mentioned above, investigating home gardens was regarded as an effective way to understand the biodiversity-related local knowledge and culture of native people in Ethnobiology and Ethnoecology. The structural and functional dynamics of home gardens could help to understand the trends in socio-economic sustainability and how these relate to ecological sustainability in local places [29]. The diversity and functions of the plants in local home garden were related closely to the local plants knowledge, which contained the understanding of local people to the plant world of their surrounding environments. Thus, the core aim of the present study was to answer the question: why local people selected these plants? And we wanted emphasize that the answer should be concluded from the local knowledge.

Specifically, the research questions of the present study are (1) how many and what plants are in the home gardens of local communities, and what are local knowledge and functions of these plants? (2) What are the most popular plants, and why they are important for local communities? (3) Why people select these plants, and what are the impact factors of the plant composition in local home gardens? The main works of the present study include (1) collecting, recording, and documenting the plants in the local home gardens, (2) visiting the home gardens and interviewing the owners of them, then recording the local knowledge of plants, and (3) documenting the local knowledge of the functions of home gardens and attempting to answer that why local people selected these plants?

## Material and methods

### Study area and communities

The study area was in Beibeng Township (Hbras-spung Shang, 背崩乡) of Motuo County. Beibeng Township was deep in the grand canyon of Yarlung Tsangpo. Almost all of the permanent population in Beibeng Township is Tsang-la people [27]. Beibeng Township was the remotest township of Motuo County, and the motor ways linked among the communities were not fully available until early 2018, when the field works of the present study were being performed in the township.

From 400 m to 3260 m, the huge variations of altitude and variety of complex topography resulted diversification of habitat types in the township. From the bottom of the canyon to the top of mountains, the vegetation was changing from tropical rain forest and subtropical evergreen board-leaved forest to alpine coniferous forest or Rhododendron shrubs. These factors gave the township rich biodiversity [15, 16].

**Fig. 1:**
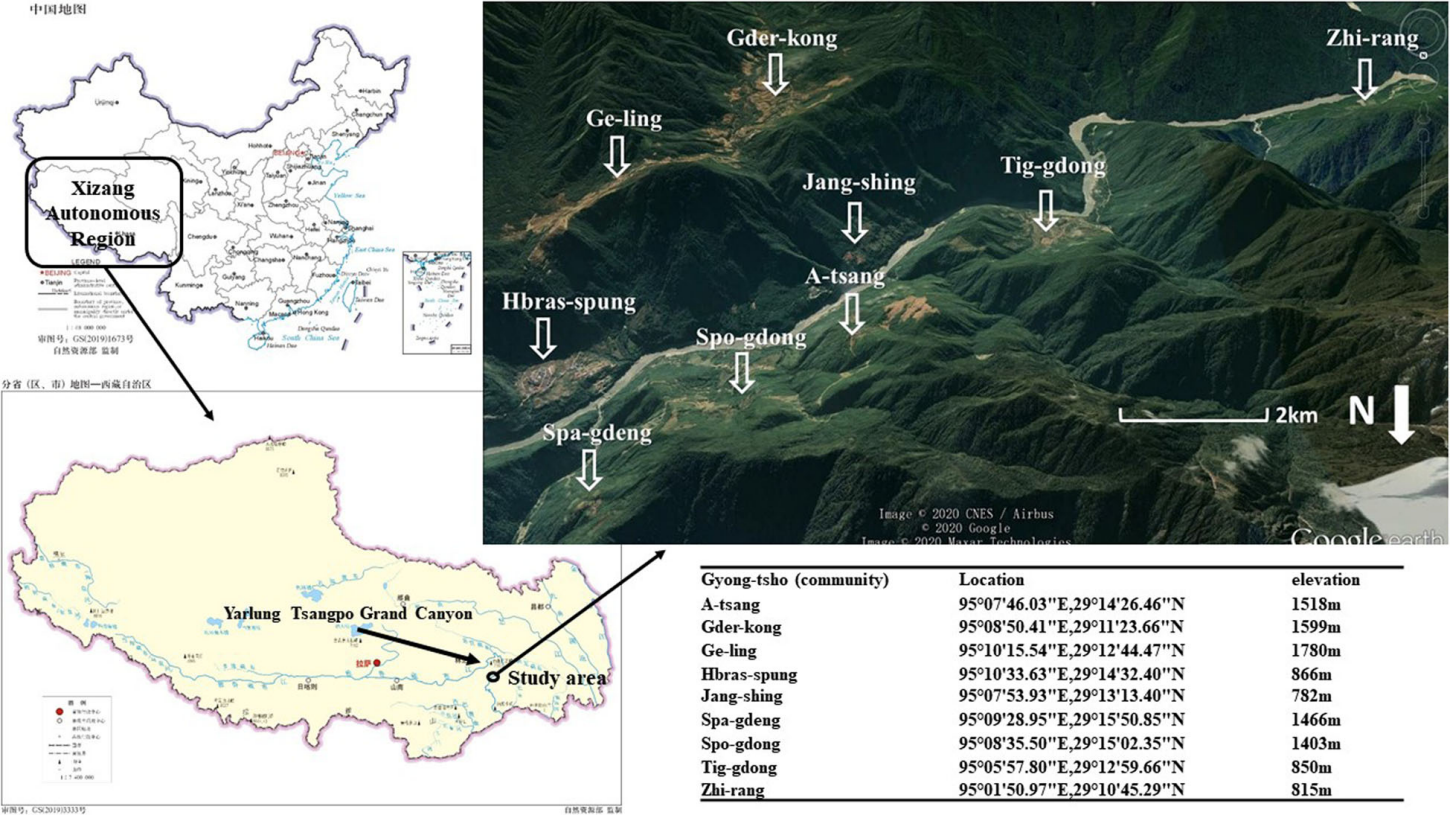
The location of study communities

The basic unit of Tsang-la community was “Gyong-tsho”. A “Gyong-tsho” usually consisted of several families related to each other. These families usually originated from the same ancestor. Therefore, in the present study, we took “Gyong-tsho” as the unit of local communities. Beibeng Township contained nine “Gyong-tshos” which located at the both sides of Yarlung Tsangpo River (Fig. 1). Each Gyong-tsho was identified as an administrative village by Chinese authority. These communities distributed in different places of the township. These communities had different elevations and surrounding vegetation.

### Field study and data collection

The field study was carried out in the nine communities of Beibeng Township from 2017 to 2018, and we visited the communities in different seasons. First, we visited the local community committee for getting field study permission. We explained our purpose to the community leaders and requested assistance from them. The assistance included providing local guides, introducing us to the community members and other necessary helps. All of our field studies were carried out with informed consent.

**Fig. 2:**
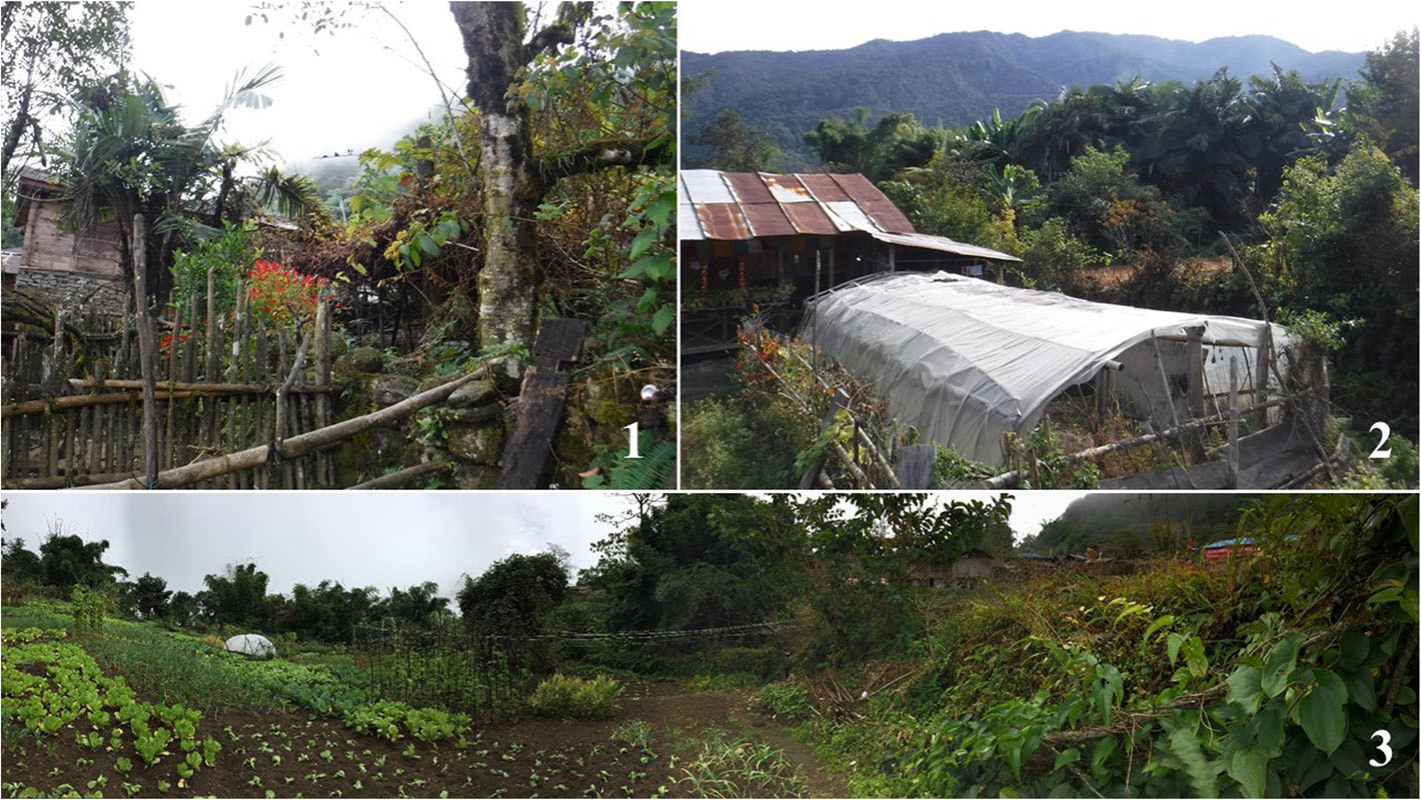
the home gardens in the Tsang-la communities in Beibeng Township. Notes: 1: A typical small “private” home garden in Spa-gdeng, the home garden belonged to just one household. 2: A typical home garden. 3: The biggest “public” home garden in Spa-gdeng, it was shared by over ten households.

**Fig. 3:**
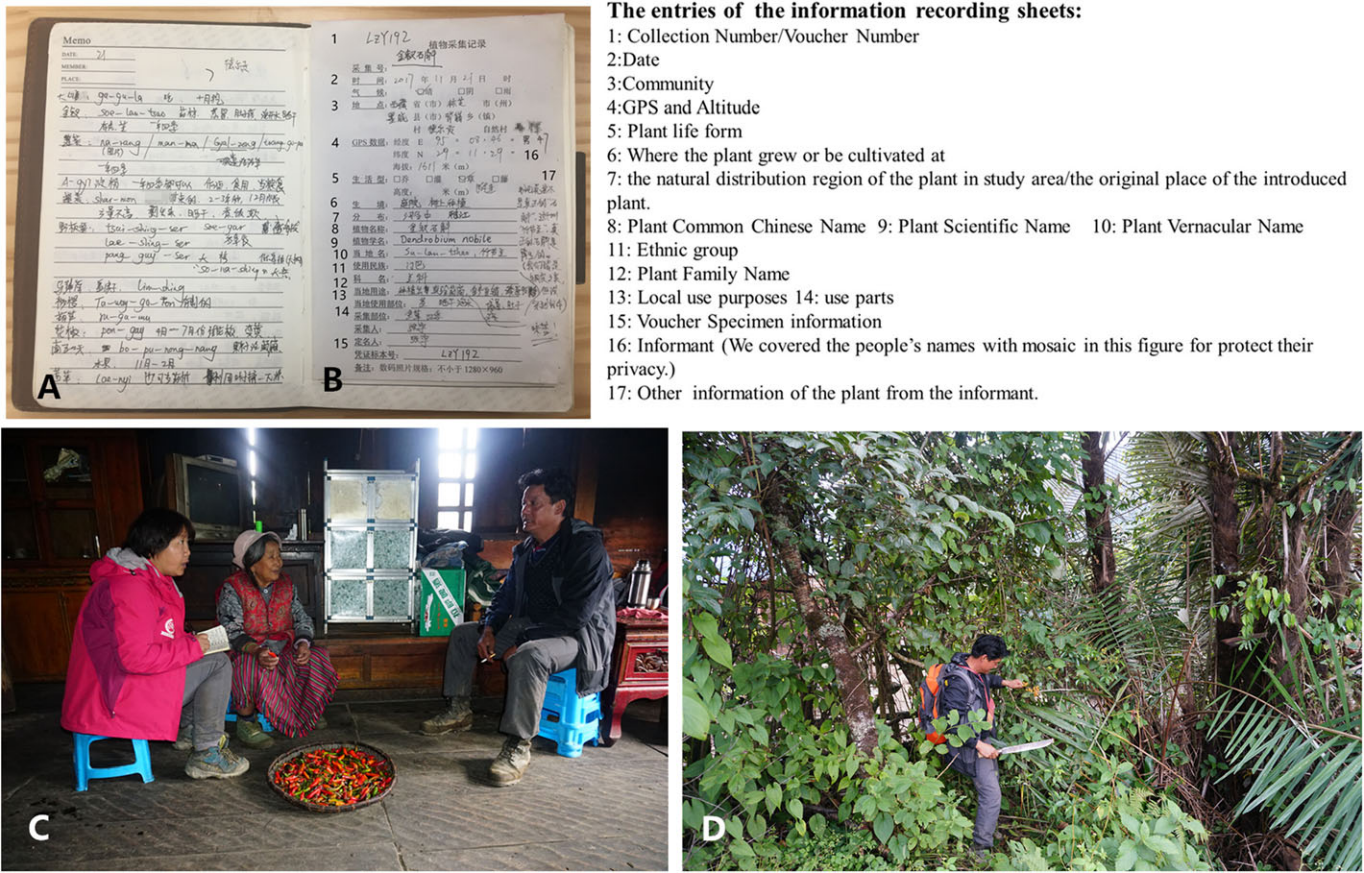
The interviews and information recording sheet example in the field study. **a** A page of portable note books. **b** An information recording sheet. **c** Interview for the detailed information of local chili. **d** The home garden owner (the same informant in c) was introducing the medicinal plant he cultivated, and he collected the fruits as research samples for us

In the present study, we set “a home garden” as a plot of land which was used to cultivate useful plants except large-scale planted crops and separated by artificial barrier with other lands in the communities (Fig. 2). Observation and semi-structure interviews were used to collect data in field study, and the semi-structure interviews were carried out with the cooperation of local guide. First, we went to visit a community member who was familiar with our local guide, and we asked him whether we could visit his/her home garden, after the guide introduced us and explained our purpose. Then, if he/she allowed us to visit the home garden, the observation and semi-structure interviews were carried out. The observation was used to record the species, sizes, and frequency of the plants in the home gardens, and the semi-structure interviews were used to get the information of the local knowledge of the plants. The local knowledge here were the knowledge provided by local people, including vernacular names, uses, features, life forms, habits, habitats, economic values, and culture significances of plants. The informants of the interviews were the owners of the home gardens. Because of the relative backwardness of the educational conditions of the study area, most of the informants could not read and write Chinese or Tibetan characters smoothly. Therefore, we summarized the detailed information collection schedule to information recording sheets and several simple questions. The semi-structure interviews were performed based on the following concise question list rather than complex questionnaires (Fig. 3).

Do you mind to introduce the plants you cultivated in your garden? And how many kinds of plants you had cultivated in your garden?

What the purpose you cultivate it? And how to use/cook/prepare it? (For one plant)

Do you mind to tell us some stories of the plant? If it is important to you/your family/your community/your religion, could you please told us more details about the importance. (For ritual plants or the plants with special or symbolic significance)

The information were recorded provisionally in portable note books, then organized into the information recording sheets with unified format, based on “one plant to one informant” as a fundamental unit (Fig. 3). We attempted to record all of the information we got from the informants to the sheets.

After we finished the study of one informant’s home gardens, we would ask him/her to introduce us to his/her neighbors or relatives in the same community. Then, we went to visit next informant. The observation and interviews would attempt to perform in consistent strategy in each home garden.

Finally, 78 home gardens were visited, and 87 home garden owners as the informants were interviewed in the field study. Because some home gardens were shared by different households and some of the informants had over one home garden, the number of visited home gardens and informants were different.

### Information documentation and plant specimen identification and preservation

All information was collected and organized from the information recording sheets, then documented to the plant catalog of the present study, based on “ethno-species” as a fundamental unit (Additional file 1). All of the ethno-species were finally identified into the scientific taxa of plants. Ethno-species was defined as a “species entity” which matched a plant product with local name, and an ethno-species was usually identified according to particular usages or features such as colors, shapes, and tastes of it by local people [28, 30]. Ethno-species was used as the primary unit of analysis in Ethnobotany, and it could reflect the local knowledge about the plants of local people in the study area.

The interviews were performed in Mandarin and Tsang-la Language, then Tsang-la language were translated to Mandarin by the local guidance. The vernacular names of plants were recorded in Tsang-la Language which was usually written in Tibetan script [31]. In this paper, we recorded the vernacular names in the Latin form of Tibetan script, due to that most of the computer systems could not display Tibetan script correctly without installing special language packs. The spelling rules of Latin form of Tibetan script could be found in the related website of omniglot.com (https://www.omniglot.com/writing/tibetan.htm) [32].

In the field study, we collected the voucher specimen and took photos to the plants. For the native and local featured plants, we collected the voucher specimen, and for the common cultivated plants such as *Brassica rapa*, *Raphanus sativus*, and *Capsicum annuum*, we took photos as the vouchers. The specimen and photos were collected with the informed consent and permission from local administrative department, and these plant specimens were identified at Herbarium of Kunming Institute of Botany (KUN). The plant specimens were identified as Flora of China and Flora of Xizang [33, 34]. The species names were checked with The Plant List [35].

### Quantitative analysis

All of the information of local use and knowledge were organized as the list of “use-report” for quantitative analysis [36]. A use-report (UR) is counted each time an informant mentions the use of an ethno-species in one of the categorized uses.

The local uses of plants were merged into 14 use categories containing fruit, ornamental plants, ritual plants, grain, wood, wine brewing, knitting, dye plants, vegetable, forage plants, spice, medicine, oil plants, and others.

#### Frequency of citation (FC) and relative frequency of citation (RFC)

Frequency of citation (FC) was used to calculation the percentage of each kind of uses and other indices. The FC is calculated as is the sum of informants that cite a use for a particular species. Relative frequency of citation (RFC) was used to show the importance of each ethno-species in the study area [36]. The values of RFC were calculated according to the formula:

$$ \mathrm{RFC}=\frac{{\mathrm{FC}}_s}{N} $$

Where FCs is the frequency of citation (the number of informants who mention the use of a species) and *N* is the total number of informants in the survey. A high RFC value for a species indicates that the species is used both frequently and by a high proportion of informants in the study area.

#### Cultural importance index

The cultural importance index (CI) was used to reflect the prevalence of use and diversity of use of each ethno-species [36]. The value of CI was the sum of proportion of informants that mention each of the use categories for a given ethno-species. The CI was defined by the following formula:

$$ \mathrm{CI}=\sum \limits_{u=1}^{\mathrm{NC}}\sum \limits_{i=1}^N\frac{{\mathrm{UR}}_{\mathrm{ui}}}{N} $$

Where URui was the total number of use-report for each use category of a given ethno-species, *N* was the total number of informants, and NC was the total number of the use categories.

#### Cultural value index

Cultural value index (CV) was developed by Reyes-Garcia et al. [37] and was the product of three factors which were calculated follow the formula:

$$ {\mathrm{CV}}_s=\left[\frac{{\mathrm{NU}}_s}{\mathrm{NC}}\right]\times \left[\frac{{\mathrm{FC}}_s}{N}\right]\times \left[\sum \limits_{u=1}^{\mathrm{NC}}\sum \limits_{i=1}^N\frac{{\mathrm{UR}}_{\mathrm{ui}}}{N}\right] $$

Factor 1: the number of use categories for the given ethno-species (NUs) divided the total number of all use categories (NC). Factor 2: the relative frequency of citation of the given ethno-species (RFC). Factor 3: the cultural importance index of the given ethno-species (CI).

The values of CV varied from 0 to FC. A high value of CV meant that most of the informants thought the ethno-species was preferred to use.

#### Jaccard Index and multidimensional scaling

The Jaccard Index (*J*), also known as the Jaccard similarity coefficient, is a statistic index for comparing the similarity and diversity between two samples [38]. In the present study, the Jaccard Index was used to compare the similarity in plant species selection among different communities in the study area. The index was calculated following the formula:

$$ \mathrm{J}\left(A,B\right)=\frac{A\cap B}{A\cup B-A\cap B} $$

Where *A* and *B* was the set of species in the home gardens of community *A* and *B*, *A* ∩ *B* was the number of the species found in both community *A* and *B* (the intersection of *A* and *B*), and *A* ∪ *B* was the total number of the species found in community *A* and *B* (the union of *A* and *B*). A high value of Jaccard Index meant that the species of the home gardens were similar between two communities.

The values of Jaccard Index were converted to the Jaccard Distance (JD) which was the complementary set of Jaccard Index.

$$ \mathrm{JD}\left(A,B\right)=1-J\ \left(A,B\right) $$

The purpose of the use of the values of the Jaccard Distance was to visualize the values of the Jaccard Index with multidimensional scaling. First, we took the values into the dissimilarity matrix. Then, we use the multidimensional scaling with ALSCAL in SPSS 19 to draw scatter plot graph, with the model of Euclidean Distance.

## Results

### Characteristics of the communities and informants

A total of 78 home gardens in the nine communities of Beibeng Township were visited (Fig. 1). The numbers and plant diversity of the home gardens in each community varied with the community population, terrain, elevation, and surrounding vegetation (Table 1). Hbras-spung was the administrative center of the township.

**Table 1:** The study communities

Gyong-tsho (community)	Elevation	Terrain	Surrounding vegetation	No. of households^*^	No. of home garden	No. of ethno-species
A-tsang	1518 m	Slope	Subtropical evergreen broad-leaved forest	35	2	12
Gder-kong	1599 m	Broad valley	Tropical mountain rain forest	50	12	80
Ge-ling	1780 m	Broad valley	Subtropical evergreen broad-leaved forest	30	7	35
Hbras-spung	866 m	Mesa	Tropical rain forest	150	40	103
Jang-shing	782 m	Slope	Tropical rain forest	25	3	23
Spa-gdeng	1466 m	Slope	Subtropical evergreen broad-leaved forest	35	4	45
Spo-gdong	1403 m	Slope	Subtropical evergreen broad-leaved forest	35	2	19
Tig-gdong	850 m	Broad valley	Tropical rain forest	130	4	17
Zhi-rang	815 m	Slope	Tropical rain forest	25	4	23

*The information was from the community committee of Beibeng Township

Eighty-seven home garden owners as informants were interviewed, and they provided 625 use reports to us. The age of informants ranged between 18 and 75 years old. Thirty-seven informants were men and 50 were women. Thirteen informants were the community cadres (Table 2).

**Table 2:** Characteristics of informants

	No. of informants
Age	
20–30	9
31–40	18
41–50	30
51–60	24
61–70	5
> 70	1
Gender	
Female	50
Male	37

### The diversity of the plants and their uses

In the present study, 196 ethno-species were collected, and they were identified into 188 Botanical taxa, which were identified two to subspecies level, 181 to species level, and 5 to the genus level, and they belonged to 160 genera and 78 families (Additional file 1). The most citied family was Asteraceae (12 species), followed by Orchidaceae (10 species), Cucurbitaceae (9 species), and Poaceae (9 species), and 44 families just only included 1 species. On genera level, the most frequent genus were Cymbidium, Dioscorea, and Solanum. Forty species were estimated and cataloged by the International Union for Conservation of Nature (IUCN), and 4 of them were categorized as endangered species. They were *Coptis teeta* (EN), *Cephalotaxus hainanensis* (EN), *Schlumbergera truncate* (VU), and *Juniperus pingii* (NT) [39]. Among them *S. truncate* were exotic cultivated flowers while others were native plants introduced from local forests. All of the plants of Orchidaceae were recorded as endangered plants in the Information System of Chinese Rare and Endangered Plants (ISCREP) [40]. One hundred and four taxa were herbaceous plants, 40 were trees, 26 were climbers, and 18 were shrubs.

The original place of plant was an important basis of ethno-species identification in local knowledge, according to the interviews to local people. Over half (122) of these ethno-species were introduced from native place or cultivated for a long time. Thirty-three ethno-species were introduced from Lhasa, 32 from other provinces of China, and 7 from foreign countries (1 from Bhutan and 6 from India).

A total of 87 home garden owners as informants were interviewed in the present study, and they provided 625 use-reports to us. These use-reports were categorized into 14 use-categories. The most cited use-category was “vegetable” (218 use-reports), followed by “ornamental plant” (108 use-reports), “medicine” (92 use-reports), and “fruit” (90 use-reports). The use-reports in these four use-categories accounted for most (81.3%) of the total numbers of use-reports. Twenty-nine ethno-species were used in over two use-categories (NU/NC > 0.14), and among them, six ethno-species were used in three use-categories (NU/NC = 0.21). No ethno-species was used in over three use-categories.

### The most popular plants

In total, there are 196 ethno-species, 15 of them had over 10 use-reports, while 85 of them had just cited 1 time (one use-report), respectively, by the informants. The ethno-species had over 10 use-reports and also had the highest values of CI and RFC. The cultural value index (CV) combined the values of CI, RFC, and NU/NC. Thus, from the values of CV of the ethno-species, the top 5 most popular ethno-species were Su-lan-tsao (*Dendrobium nobile*, CV = 0.0064), Sa-ga (*Zingiber officinale*, CV = 0.0044), Soe-lu (*Capsicum annuum*, CV = 0.0028), Snying-pa (*Citrus medica*, CV = 0.0020), and Kham-pu (*Prunus persica*, CV = 0.0019) (Table 3). These plants were all cultivated for a long time by local people.

**Table 3:** The top 5 popular ethno-species

Vernacular name	Scientific name	Family	Origin	Life form	Use part	Use	Use category
Su-lan-tsao; 竹节兰, 石斛	*Dendrobium nobile* Lindl.	Orchidaceae	Native	Herb	Stem and flowers	It was collected and cultivated as economic useful plant in local tropical regions. It was the original plant of traditional Chinese medicine “Shi Hu (石斛)” and traditional Tibetan medicine “bu-shes-tse.” It was also cultivated as ornamental plants in home gardens.	Ornamental plants; medicine; others
Sa-ga; 生姜	*Zingiber officinale* Roscoe	Zingiberaceae	Native	Herb	Rhizome	The rhizomes were used as vegetable, spice and medicine. The rhizomes were used to treat cold in local medicine.	Medicine; vegetable; spice
Soe-lu; 本地辣椒	*Capsicum annuum* L.	Solanaceae	Native	Herb	Fruit	Local *Capsicum annuum* had several cultivated varieties, different varieties were used for different purposes. And all of them were divided into two ethno-species based on the original places. Soe-lu is native.	Vegetable; spice; medicine
Snying-pa; 墨脱大柠檬	*Citrus medica* L.	Rutaceae	Native	Tree	Fruit	The fruits were local specialty “Motuo Da Ningmeng (墨脱大柠檬)” and used as traditonal Chinese Medicine “Xiang Yuan (香橼).”	medicine
Kham-pu; 桃	*Prunus persica* (L.) Batsch	Rosaceae	Native	Tree	Fruit	It was cultivated for harvest fruits. Kham-pu was important in traditional Tibetan culture which symbolized auspiciousness.	Fruit

### Comparison of the species among the communities

**Fig. 4:**
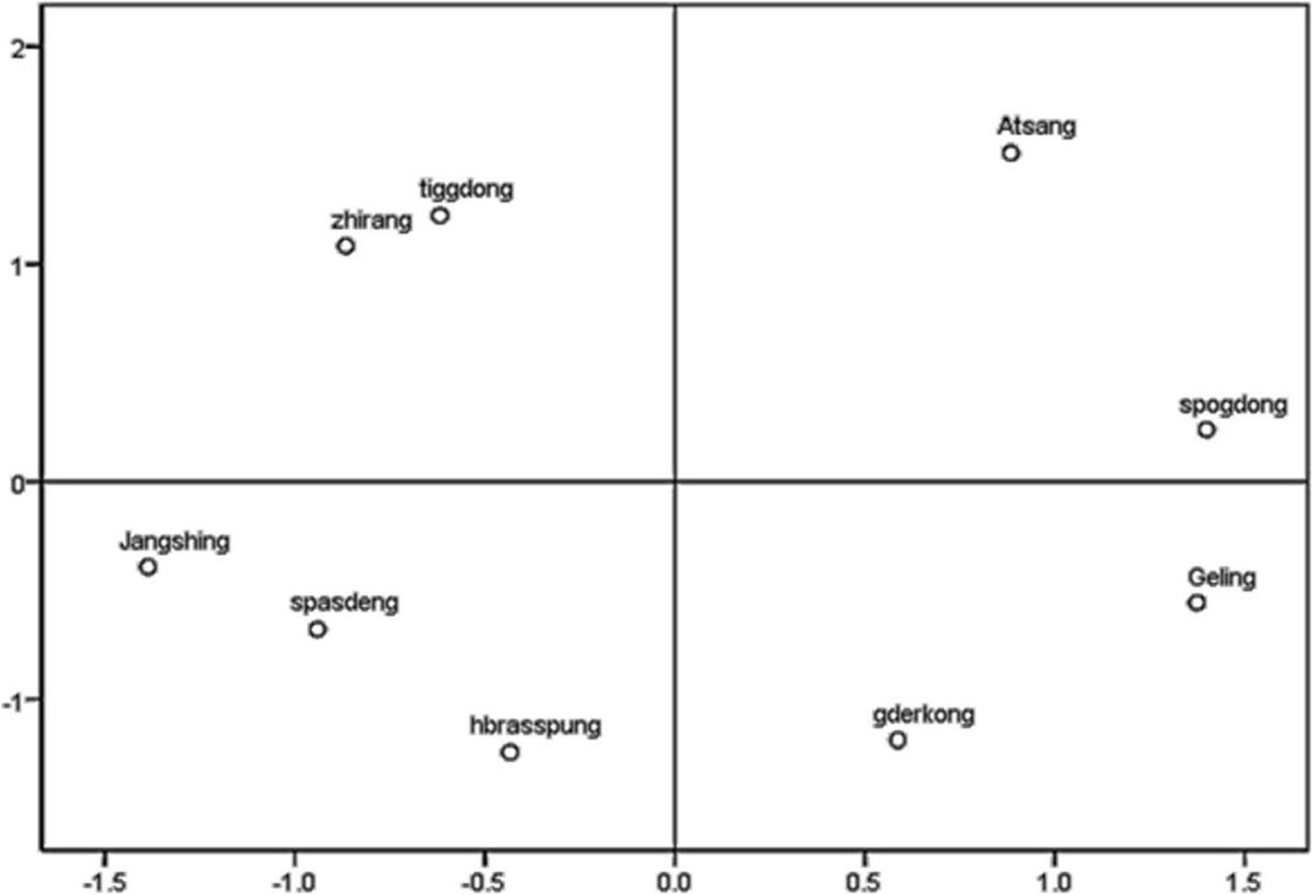
The multidimensional scaling (ALSCAL) based on Jaccard Distance

The results of Jaccard Index were in Table 4 and Fig. 4. The highest value of Jaccard Index is 0.236 (Hbras-spung and Gder-kong), followed by 0.194 (Hbras-spung and Spa-gdeng), and the lowest value of Jaccard Index is 0.018 (Ge-ling and Zhi-rang). From the results of multidimensional scaling (ALSCAL) (Fig. 4), we could divide the communities into four groups, with their positions in the four quadrants of Fig. 4.

**Table 4:** The Jaccard Index of comparison the similarity in plant species selection among different communities in the study area

	A-tsang	Gder-kong	Ge-ling	Hbras-spung	Jang-shing	Spa-gdeng	Spo-gdong	Tig-gdong	Zhi-rang
A-tsang	1.000	0.045	0.068	0.036	0.029	0.056	0.107	0.036	0.094
Gder-kong	0.045	1.000	0.162	0.236	0.120	0.157	0.100	0.090	0.096
Ge-ling	0.068	0.162	1.000	0.095	0.036	0.143	0.149	0.040	0.018
Hbras-spung	0.036	0.236	0.095	1.000	0.156	0.194	0.061	0.101	0.105
Jang-shing	0.029	0.120	0.036	0.156	1.000	0.153	0.050	0.111	0.095
Spa-gdeng	0.056	0.157	0.143	0.194	0.153	1.000	0.123	0.107	0.172
Spo-gdong	0.107	0.100	0.149	0.061	0.050	0.123	1.000	0.091	0.024
Tig-gdong	0.036	0.090	0.040	0.101	0.111	0.107	0.091	1.000	0.111
Zhi-rang	0.094	0.096	0.018	0.105	0.095	0.172	0.024	0.111	1.000

## Discussion

### The important plants in home gardens

**Fig. 5:**
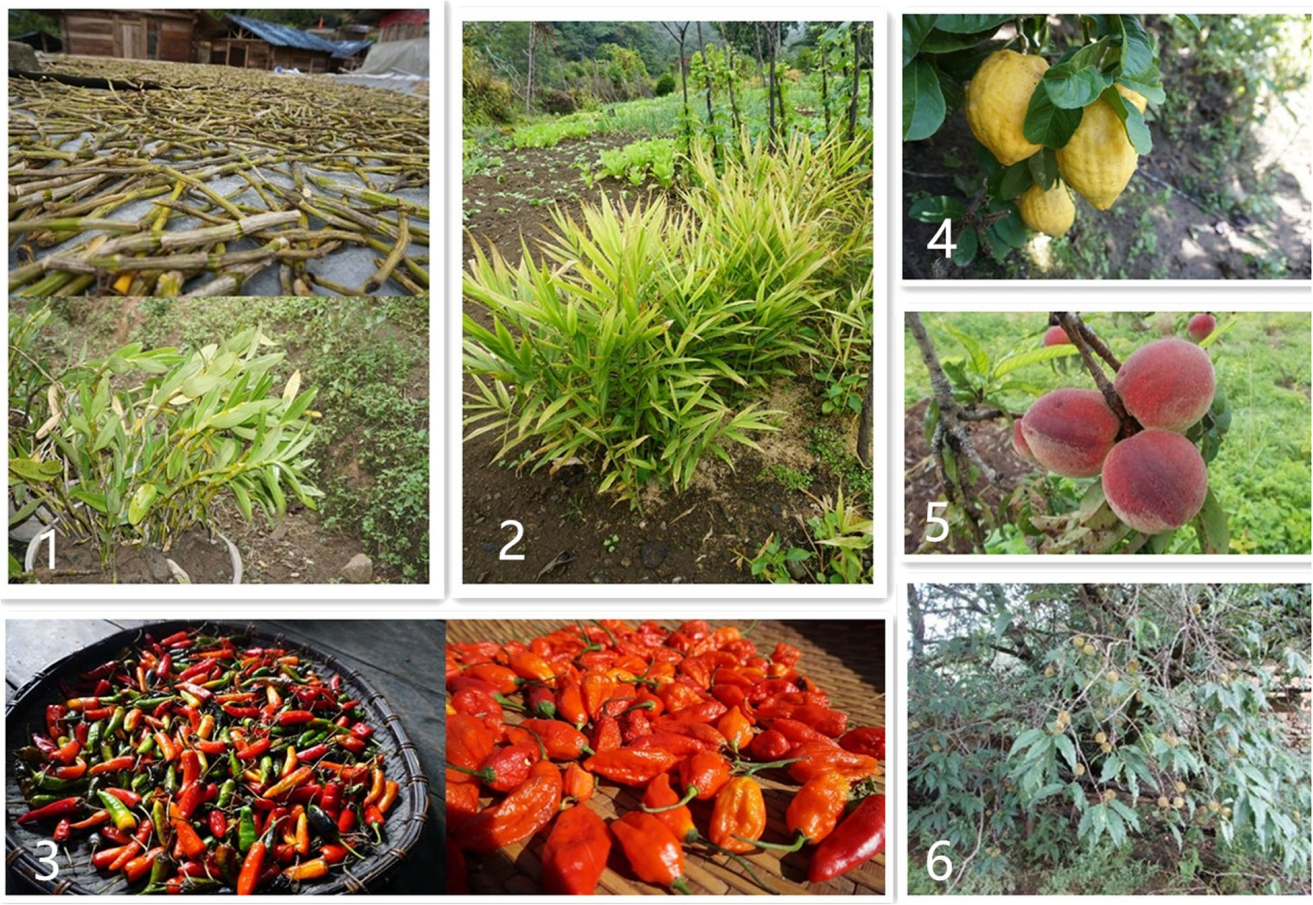
The top 5 important plants in home gardens. Notes: **1** *Dendrobium nobile*. **2** *Zingiber officinale*. **3** *Capsicum annuum*. **4** *Citrus medica*. **5** *Prunus persica*. **6** *Prunus mira*

Based on the quantitative analysis, we found that some plants were important. These plants were cultivated commonly in the home gardens or cited frequently by the informants (Fig. 5).

Su-lan-tsao (*Dendrobium nobile*) was the most frequently cited and cultivated plants in the home gardens. The plant was cultivated as featured product in the tropical and subtropical regions of Motuo County. *D. nobile* was the original plant of the Traditional Chinese Medicine “Shi-hu (石斛)” and the Traditional Tibetan Medicine “Bu-shes-tse” [41, 42], and it could be sold for a high price in traditional medicine markets. Thus, *D. nobile* was popular to cultivated by local people for selling. Besides, another purpose of Tsang-la people to cultivate the plant was for decorating home gardens because of its beautiful flowers.

Sa-ga (*Zingiber officinale*) was multipurpose in local communities. The tender rhizomes of *Z. officinale* were seasonal vegetable in springs, and the mature rhizomes which were rich in aromatic oil were used as spice in cooking. Local Tsang-la people also used the rhizome as medicine to treat cold. *Z. officinale* was the original plant of Traditional Tibetan Medicine “Ga-kya” which was used to treat digestion problems and the complications of “rlung (wind)” and “bad-gan (fluid)” diseases [42].

*Capsicum annuum* was the most important spice flavor in typical Tsang-la daily cuisines, and it was used in almost every local dish. Two ethno-species of *Capsicum annuum* were cultivated by Tsang-la people in the study area. The two ethno-species were identified based on the pungency level and origin places. One was named as “Soe-lu” which had temperate pungency, and the other one was named as “Bon-ga-soe-lu” which had strong pungency. “Soe-lu” was native while “Bon-ga-soe-lu” was introduced from India. We found that the morphological feature of “Bon-ga-soe-lu” was similar with “Bhut Jolokia (India’s ghost pepper)” of India [43].

Nying-pa (*Citrus medica*) was common in local home gardens. Tsang-la people cultivated it for collecting fruits as medicine, traditionally. Tsang-la people collected the fruits of *C. medica*, then sliced up and dried the fruits in shade. The dried fruits were used as herbal tea to treat cold in local Tsang-la medicine. Local people named *C. medica* as “Motuo Da Ningmeng (墨脱大柠檬, Big Lemon of Motuo County)” in Chinese language, because the smell of its fruits was very similar to lemon. Besides, *C. medica* was the origin plant of Traditional Chinese Medicine “Xiang-yuan (香橼)” [40], so local people also collected its fruits and sold to traditional druggists.

Kham-pu (*Prunus persica*) was another highly frequent ethno-species in local home gardens. *P. persica* was cultivated as fruiter. Besides, Kham-pu was an important ritual plant in traditional culture which symbolized auspiciousness in Xizang District [44]. Another *Prunus* species, *P. mira* was also called as Kham-pu by local people, but local people thought it was not the same ethno-species with *P. persica* because the species was used for other purpose and not harvesting fruits. *P. mira* was a common wild plant in Yarlung Tsangpo Canyon, and local people introduced it into home gardens as rootstocks for grafting *P. persica*.

### Local knowledge of the identification of ethno-species

The diversity of ethno-species could indicate the local knowledge of understanding plants. In the study area, Tsang-la people identified the ethno-species from the use purpose and origin of plants. The ethno-species could mostly match to the botanical species, but sometimes under-differentiated or over-differentiated to botanical species [28, 30]. For example, *Brassica rapa* (total UR = 31) was the most frequent botanical species in the present study. *Brassica rapa* was identified as four ethno-species by local people, according to the differences of their cooking methods and morphological characteristics among them. For another example, Tsang-la people divided the plants of genus *Musa* into two ethno-species as “Tsun-ma-lai-su,” of which the tender shoots and flowers were edible as vegetable, and “A-ni-lai-su,” of which the fruits were edible as banana, although we found that at least four botanical species of the genus were used by local people. Further, Tsang-la people divided the local cultivated populations of “A-ni-lai-su” into “Krung-go-a-ni-lai-su,” which was native or introduced from other regions of China, and “Ni-ra-a-ni-lai-su,” which was introduced from India, based on their origin places.

### The important functions of the local home gardens

#### The self-service “food supermarkets” and “herb shops” of local people

Home gardens not only had high plant diversity, but also many functional plants including edible and medicinal plants [1, 2].

From the previous studies, food plants were the most frequently cited in most of the home gardens through the world [1–3]. The results of our study were no exception. The plants used as vegetable (218 use-reports) were cited most frequently in the home gardens in the present study. The edible plants could provide carbohydrates, proteins, vegetable oil, vitamins, and minerals [3, 9, 45, 46]. Our previous study in Yunnan Province of China showed that the edible plants in home gardens and wild could help people to fight against famine [47]. Nepali experts also found that home gardens were important to food supply in remote mountain area of Nepal [10].

Asteraceae was the most frequently cited families containing 12 botanical species in the present study, and 7 of them were used vegetables. The plants of genus *Dioscorea* and *Solanum* were cited frequently; these species were used as food and medicine.

Thirty-three ethno-species were cultivated for medicinal uses. These medicinal plants were used to treat common ailments by local people, and some of them were sold to the druggists. The previous studies in Yunnan Province of China and Chiang Mai of Thailand also showed similar results [5, 8].

Home gardens were also used as “crops seedling bases” by local people. Rice was the staple crop in the low altitude regions of Beibeng Township (the Tsang-la language “Hbras-spung” meant “rice field”). Local people raised the rice seedlings in the home gardens then planted the seedlings into the field in every spring. Beside rice seedlings, the seedlings of vegetables, fruit trees, and some medicinal plants were also raised in home gardens. Local people usually built small greenhouses for crop seedling in their home gardens.

Besides, the home gardens were also the bases of local people for introducing alien plants from other places, and most of these plants were edible or medicinal plants. These plants enriched the food tables and herb chests of local people.

The surplus products from home garden could be sold in local small markets. Moreover, some plants, such as *Dendrobium nobile* and *Camellia sinensis*, were especially cultivated for selling. Thus, local home garden could be seen as local “supermarkets” or “shops” for increasing incomes of local people. According to some previous studies, the farming in home gardens could be as one of the ancillary incomes for local people especially women [1–3, 48–50]. In the present study, we found that most of the local home gardens were as if managed by women, and also, the number of female informants was actually more than male informants. But we could not conclude that women were more important than men in the farming and management of local home gardens, because the detailed related information were not enough in the present study. We need to collect more information in future field researches.

#### The base of local native endangered plants conservation and sustainable use

Home gardens may contribute to the conservation of native species [51]. Four species in the home gardens were designed as endangered plants by IUCN, and three of them including *Coptis teeta* (EN), *Cephalotaxus hainanensis* (EN), and *Juniperus pingii* (NT) were native species [39]. All of the plants of Orchidaceae were recorded as endangered plants in the Information System of Chinese Rare and Endangered Plants (ISCREP) [40]. These plants were important for local medicine, ornament, and religion (Table 5). Local people introduced them from forests and cultivated and bred them carefully in their home gardens. Some previous studies indicated that this phenomenon could help conserve the local endangered useful plants, and it was an efficient way to rare plant resources sustainable uses [1–4, 51–54]. Besides, the previous studies showed that local home gardens were important in conserving the regional biocultural heritage of local economic plant resources [55–57]. It was worth mentioning that local people told us that the reason why they cultivated these endangered plants was for increasing the available resources of these plants. “These plants are extremely rare,” a home garden owner told us, “if we always collect these plants in the forest rather than attempt to cultivate them, they will disappear.”

**Table 5:** The endangered plant species in the home gardens

Vernacular name	Science name	Family	Endangered levels	Origin	Life form	Use part	Use category
Shug-pa	*Juniperus pingii* W.C.Cheng ex Ferré	Cupressaceae	NT	Native	Shrub	Leaves and seeds	Ritual plants; medicine
Mong-nang	*Schlumbergera truncata* (Haw.) Moran	Cactaceae	VU	Lhasa	Herb	Flowers	Ornamental plants
Ton-tsa	*Coptis teeta* Wall.	Ranunculaceae	EN	Native	Herb	Rhizome	Medicine
Bras-shing	*Cephalotaxus hainanensis* H.L.Li	Taxaceae	EN	Native	Tree	Stem	Wood; ritual plants
Mong-nang	*Arundina graminifolia* (D.Don) Hochr.	Orchidaceae	LC	Native	Herb	Flowers	Ornamental plants
Mong-nang	*Calanthe* sp.	Orchidaceae	LC	Native	Herb	Flowers	Ornamental plants
Lan-tshao	*Cymbidium eburneum* Lindl.	Orchidaceae	DD	Native	Herb	Flowers	Ornamental plants
Lan-tshao	*Cymbidium elegans* Lindl.	Orchidaceae	EN	Native	Herb	Flowers	Ornamental plants
Lan-tshao	*Cymbidium goeringii* (Rchb.f.) Rchb.f.	Orchidaceae	DD	Native	Herb	Flowers	Ornamental plants
Lan-tshao	*Cymbidium iridioides* D.Don	Orchidaceae	VU	Native	Herb	Flowers	Ornamental plants
Lan-tshao	*Dendrobium densiflorum* Lindl.	Orchidaceae	VU	Native	Herb	Flowers	Ornamental plants
Su-lan-tsao; 竹节兰, 石斛	*Dendrobium nobile* Lindl.	Orchidaceae	VU	Native	Herb	Stem and flowers	Ornamental plants; medicine; others
Lan-tshao	*Dendrobium williamsonii* Day & Rchb.f.	Orchidaceae	EN	Native	Herb	Flowers	Ornamental plants
Shing-lan-tshao	*Vanda cristata* Wall. ex Lindl.	Orchidaceae	EN	Native	Herb	Flowers	Ornamental plants

*Dendrobium nobile* was the most frequent plant in local home gardens of Tsang-la people, and it was important sample commodity in health product markets. However, it was identified as endangered species in ISCREP [40], and the wild population commercial collection and cross-border trade of the species was illegal in China [58]. The artificial cultivation of medicinal plants was considered as an efficient way to reduce the collection from wild population. The function for biodiversity conservation of the local home gardens could coincide with the concept of the “Biodiversity conservation through use” [59]. Home gardens could emerge as an effective means for both economic well-being and biodiversity conservation [60].

#### The important part of beautiful homes

Home gardens provided many beautiful plants for decorating daily lives, and many of these plants were with great potentials to be developed as ornamentals in modern Horticulture [61]. In the present study, we recorded 49 ethno-species which were cultivated as ornamental plants, and 32 of them were called as “Mong-nang” by local people. “Mong-nang” meant “beautiful flower” in Tsang-la language. In Tsang-la communities, “Mong-nangs” were important decorations in home gardens and balconies. Local people collected ornament plants from native forests and other places, then introduced to their home gardens. The informants told us that a big part of the “Mong-nangs” (we had recorded 29 ethno-species) were introduced from Lhasa, the center city of Xizang Autonomous Region, and they got the seedlings in Lhasa and secured them carefully on the long way home. The informants claimed that beautiful flowers were essential in their lives. Interestingly, local people did not seem to know the Tsang-la name of each “Mong-nang,” but called them collectively as “Mong-nang.” It might because that most of these plants were introduced form other places, they had no Tsang-la names. According to the study on traditional culture of the ethnic groups in Tibetan Plateau, the ideal homes should be surrounded by beautiful gardens with many blooms [62].

Interestingly, the ornamental plants of Orchidaceae especially Genus *Cymbidium* were cited frequently and cultivated carefully in the home gardens by local people. Local people named them as “Lan-tshao.” We inferred that the name was from Mandarin “Lan-cao (兰草).” The ornamental Orchidaceae plants were important in traditional Chinese culture, and they could fetch a good price in flower markets [63].

### The impact factors of the plant diversity and arrangement in home gardens

From the results of multidimensional scaling (ALSCAL) (Fig. 4), we could divide the communities into four groups with their positions in the four quadrants of Fig. 4. We found that all of the communities in quadrants 1 and 2 were located on the west side of Yarlung Tsangpo River while most of the communities except Spa-gdeng in quadrants 3 and 4 were located on the east side of the river. And the altitudes of all of the communities in quadrants 1 and 4 were over 1000 m while the altitudes of most of the communities except Spa-gdeng in quadrants 2 and 3 were below 1000 m.

Spa-gdeng shared many home garden plants with the low altitude communities on the east side of Yarlung Tsangpo River especially Hbras-spung (*J* = 0.19, the second-highest of the values of Jaccard Index), although it was a high altitude community located on the west side of the river. Why Spa-gdeng was so different with other communities? We inferred that it might because Spa-gdeng was near the main road, which provided many chances for the people of Spa-gdeng to exchange plants and information with people from other places.

Therefore, we inferred that the altitude might be the most important impact factor of the plant diversity and composition of home gardens, and the traffic conditions also impact the plant diversity and composition. Similarly, a previous study in Africa indicated that the major factors of the plant diversity in home gardens were geographical distance between sites and differences in altitude of farms [64]. Furthermore, the previous studies in central Himalaya and Nicaragua showed that the factors of the plant composition in home gardens included climate, local social-economic condition, personal preference, and cultural background of the owners [65–67]. In the present study area, because of the huge altitude variations in the relatively small region, the altitude might be the principal factor of local climate conditions of different communities.

Other factors, such as local terrain, also impacted the plant diversity of home gardens. Hbras-spung and Gder-kong not only had the most number of home gardens but also the highest diversity of plants in the home gardens. We inferred that the possible reason was that the two communities located at mesas or broad valleys, and they had more available flat fields for cultivating than other communities which usually located at rough terrain places. One home garden usually belonged to one household, in general. But in the local communities, we found that some home gardens were not private but shared by several households. Because of the limited available flat fields, the households usually shared home gardens, especially in the communities located at rough terrain places, such as Spo-gdong, Spa-gdeng, and Jang-shing. Thus, we categorized the local home gardens into “private” and “public” home gardens (Fig. 2). A typical example was that, in Spa-gdeng, most of the households shared two big public home gardens, while just few households could have their own private small home gardens. We found that the diversity of public home gardens was much richer than private home gardens, because the public home gardens were larger. A previous study in Kumaun Himalaya Region suggested that large home gardens are more efficient than the small home gardens and are ecologically, socially, and economically diversified [68].

### Issues of the impaction of the old houses reconstruction to the local home garden

Dig-gdong was a large community included 130 households, and the community had enough flat fields. But the big project of old houses reconstruction was in progress in the community, and most of the old facilities including houses and home gardens were dismantled. Thus, only just four survive home gardens were recorded. Interestingly, we recorded the largest number of home gardens and richest diversity of plants in Hbras-spung, of which the old house reconstruction projects had been finished several years ago. Because the information of the plants in the home gardens in Hbras-spung before the old houses reconstruction projects was not available, we could not do further analysis. Therefore, it was difficult to know how the projects impact the diversity and composition of plants in the home gardens of local communities.

With the rapid economic development, the old houses reconstruction had implemented in more and more rural communities of developing regions [26]. The related researches in Southeast Asia showed that recent socio-economic changes of local home gardens were converting subsistence-oriented home gardens into commercial ones [69]. However, few studies focused on that how the old houses reconstruction change the landscape patterns including the plant composition of home gardens of rural communities. Future researches should pay more attention to the issues.

## Conclusion

In remote areas such as the Yarlung Tsangpo Grand Canyon, the plants in home gardens are important sources of plant products such as foods, herbal medicines, and fibers to support daily lives. The local home gardens in Tsang-la communities had high diversity of plants, and these plants provided many functions and services to support daily lives of local people. A total of 78 home gardens in the 9 communities of Beibeng Township were visited, and 196 ethno-species were collected. These ethno-species were identified into 188 Botanical taxa, which were identified two to subspecies level and 181 to species level and five to the genera level, and they belonged to 160 genus and 78 families. The top 5 important plants were Su-lan-tsao (*Dendrobium nobile*), Sa-ga (*Zingiber officinale*), Soe-lu (*Capsicum annuum*), Snying-pa (*Citrus medica*), and Kham-pu (*Prunus persica*), according to the CV values. The most citied family was Asteraceae, followed by Orchidaceae, Cucurbitaceae, and Poaceae. A total of 87 home garden owners as informants were interviewed in the present study, and they provided 625 use-reports to us. These use-reports were categorized into 14 use-categories. The most citied use-category was “vegetable,” followed by “ornamental plant,” “medicine,” and “fruit.” We inferred that the altitude might be the most important impact factor of the plant diversity and composition of home gardens, and the traffic conditions also impact the plant diversity and composition. Other factors such as local terrain also impact the plant diversity and composition of home gardens.

Local plant knowledge, including the features, life forms, habits, habitats, and use values of plants, were the summary of the understanding of local people to their surrounding plant worlds. Local people got the information of plants by observation, intuition, experience, and trial, then summarized them as local knowledge by their own language. Finally, local people selected appropriate plants to cultivate and manage in their home gardens under the guidance of the local plant knowledge. These plants which had various functions provided necessary products and services to support the daily lives of local people. That is the answer to the question “why local people selected these plants?”

## Supplementary information

**Additional file 1.**

## Data Availability

Please contact the corresponding author for data requests.
